# The usefulness of contrast during exercise echocardiography for the assessment of systolic pulmonary pressure

**DOI:** 10.1186/1476-7120-6-51

**Published:** 2008-10-13

**Authors:** Luis R Lopes, Maria J Loureiro, Rita Miranda, Sofia Almeida, Ana R Almeida, Ana Cordeiro, Carlos Cotrim, Manuel Carrageta

**Affiliations:** 1 Cardiology Department, Hospital Garcia de Orta, Av. Torrado da Silva, Pragal 2801-951 Almada, Portugal; 2 Reumatology Department, Hospital Garcia de Orta, Av. Torrado da Silva, Pragal 2801-951 Almada, Portugal

## Abstract

**Background:**

The systolic pulmonary artery pressure (PAPs) can be accurately estimated, non-invasively, using continuous-wave Doppler (CWD) ultrasound measurement of the peak velocity of a tricuspid regurgitant (TR) jet.

However, it is often difficult to obtain adequate tricuspid regurgitation signals for measurement of PAPs, what could lead to its underestimation. Therefore, utilization of air-blood-saline contrast has been implemented for the improvement of Doppler signal in several clinical contexts.

It is now recommended in the evaluation of patients with pulmonary hypertension. Physical activity is severely restricted in patients with PAH, being exertional dypnea the most typical symptom. Exercise stress echo-Doppler imaging allows assessment of the response to exercise. It is an excellent screening test for patients with suspected PAH. Our purpose was to evaluate the value and accuracy of agitated saline with blood contrast echocardiography, in the improvement of the Doppler signal, to quantify PAPs during treadmill exercise-echocardiography.

**Purpose:**

To evaluate the value of contrast echocardiography, using agitated saline with blood, in the improvement of the Doppler signal used to quantify the pulmonary artery systolic pressure during exercise.

**Methods:**

From a total of 41 patients (pts), we studied 38 pts (93%), 35 women, aged 54 ± 12 years-old. 27 with the diagnosis of systemic sclerosis, 10 with history of pulmonary embolism and one patient with a suspected idiopathic PAH, who were referred to the Unity of Heart Failure and Pulmonary Hypertension for screening of PAH. According to the Unity protocol, a transthoracic echocardiogram was made, in left decubitus (LD), with evaluation of right ventricle-right atria gradient (RV/RAg). A peripheral venous access was obtained, with a 3-way stopcock and the patients were placed in orthostatism (O), with a new evaluation of RV/RAg. Exercise echocardiography (EE) was begun, with evaluation of RV/RAg at peak exercise (P) and afterwards agitated saline (8 cc with 1 cc of air and 1 cc of blood) was injected, followed by a new evaluation of RV/RAg (PC) and then the interruption of the EE. Pulmonary Hypertension was diagnosed when RV/RAg at the end of the exercise was superior to 40 mmHg.

**Results:**

The quality of Doppler signal was deteriorated in 5 pts, maintained in 6 pts and improved in 26 pts, with the use of contrast. In one patient, an interventricular septal defect was diagnosed. In 6 pts, a Doppler signal was only obtained with the use of contrast. In 15 pts, a RV/RAg superior to 40 mmHg was only obtained with the use of contrast. Of these, 9 have already been submitted to right heart cathetherism, that confirmed the diagnosis of pulmonary hypertension in 5 of them (56%). RV/RAg (P) was 44 ± 11 mmHg and RV/RAg (PC) was 54 ± 11 mmHg, p < 0,001.

**Conclusion:**

1. The method is applicable in a large number of patients. 2. RV/RA gradients obtained at peak exercise are higher with the use of contrast, and the clinical meaning of this difference should be evaluated in a larger number of pts submitted to right heart cathetherism. The high number of false positives should lead to a higher diagnostic threshold. 3. This method seems to have relevant clinical value in the diagnosis of pulmonary arterial hypertension.

## Introduction

The systolic pulmonary artery pressure (PAPs) can be accurately estimated, non-invasively, using continuous-wave Doppler (CWD) ultrasound measurement of the peak velocity of a tricuspid regurgitant (TR) jet. The systolic pressure gradient (delta P) between right ventricle and right atrium is calculated by the modified Bernoulli equation (delta P = 4V2). Adding the transtricuspid gradient to the mean right atrial pressure – estimated by the assessment of the righ atrial size, inferior vena cava diameter and inferior vena cava inspiratory collapse – gives predictions of right ventricular systolic pressure that correlated well with right heart catheterization values, in several studies made in multiple clinical contexts, both in congenital and acquired heart disease [1-5].

Noninvasive estimation of pulmonary arterial pressure is important for haemodynamic monitoring of patients with heart disease, obviating the need for repeated catheterization.

However, it is often difficult to obtain adequate tricuspid regurgitation signals for measurement of PAPs, what could lead to its underestimation. Therefore, utilization of air-blood-saline contrast has been implemented for the improvement of Doppler signal in several clinical contexts. It is now recommended in the evaluation of patients with pulmonary hypertension [6-8].

Physical activity is severely restricted in patients with PAH, being exertional dypnea the most typical symptom. The parameters obtained at rest poorly correlate with symptoms or exercise response and have modest prognosis significance, in contrast with the 6 minute walking test or cardiopulmonary exercise test, that assess exercise intolerance.

Exercise stress echo-Doppler imaging allows assessment of the response to exercise of left and right ventricular function, transvalvular and prosthetic valve gradients, and right ventricular systolic pressure. It has substantial clinical use in the evaluation of patients with equivocal evidence of mitral stenosis, suspition of dynamic mitral insufficiency or prosthetic valve dysfunction, aortic stenosis and impaired left ventricular systolic function, diseases associated with pulmonary hypertension or cor pulmonale, and among patients with dyspnea of unknown origin [9].

It is an excellent screening test for patients with suspected PAH: in asymptomatic individuals at risk it may unmask abnormal increases in pulmonary artery pressures during exercise, corresponding to early stage disease [9,10].

It was already demonstrated that exercise echocardiography can be used to determine the extent of pulmonary vascular damage in patients with chronic obstructive pulmonary disease, by showing the presence of exertional pulmonary hypertension in subjects with normal PAPs at rest [11].

It is also useful in patients with connective tissue disease to screen for lung involvement by identifying exertional pulmonary hypertension, and in patients with established pulmonary hypertension to choose and monitor the effects of therapeutic interventions on the PAPs [11].

We believe that treadmill exercise is more closely related, compared with bycicle exercise, to the daily physical activity demands. Although the feasibility of treadmill stress echocardiography is described as lower in most of the publications, there isn't, at the moment, any study that makes a direct comparison between the two types of exercise stress echocardiography.

Our purpose was to evaluate the value and accuracy of agitated saline with blood contrast echocardiography, in the improvement of the Doppler signal, to quantify PAPs during treadmill exercise-echocardiography.

## Methods

### Population

From 41 patients, we studied 38 patients (93%) – 35 women (92%) – aged 54 ± 11 years-old. Three patients were excluded due to poor quality images. Twenty-seven patients had a diagnosis of systemic sclerosis, 10 patients had pulmonary embolism history and one patient had a suspected idiopathic pulmonary hypertension (PAH) without TR. They were referred to the Unity of Heart Failure and Pulmonary Hypertension for screening of PAH.

### Exercise echocardiography

A Philips Sonos 7500 ^® ^echocardiograph was used. All exams were digitally recorded.

According to the Unity protocol, a rest transthoracic echocardiogram was made first, in left decubitus (LD), which included evaluation of right ventricle-right atria gradient (RV/RAg). A peripheral venous access was then obtained, with a 3-way stopcock, and the patients were placed in orthostatism (O), with a new evaluation of RV/RAg.

Exercise echocardiography (EE), performed according to the European Association of Echocardiography guidelines [12], was begun (additional file 1), with evaluation of RV/RAg at peak exercise (P) and afterwards agitated saline contrast (8 cc with 1 cc of air and 1 cc of blood) was injected, followed by a new evaluation of RV/RAg (PC) – still in orthostatism- and the interruption of the EE (additional files 2 and 3). Pulmonary Hypertension was diagnosed when RV/RAg at the end of the exercise was superior to 40 mmHg (according to previous reports on normal values of PAPs during exercise in healthy individuals).

Heart rate, systemic blood pressure and EKG were monitored throughout the exam.

### Right heart catheterization

The patients considered to have PAH by echocardiography were submitted to right heart catheterization, with a Swan-Ganz catheter.

According to the published guidelines, PAH was defined by a mean PAP > 25 mmHg at rest or > 30 mmHg with exercise [13].

### Statistical analysis

We compared the value of peak exercise RV/RAg, before and after injection of contrast, with a student t test.

Of the patients with echocardiographically diagnosed PAH, we compared the results of the non-invasive and invasive evaluation.

We also analyzed and described the number of patients with improved, equal and worsened CWD signal of the TR jet, after injection of contrast.

## Results

The general echocardiographic parameters of the population are characterized in table 1.

**Table 1:** General echocardiographic characterization of the population

**Echocardiographic parameters**	
Left ventricle end-diastolic diameter (M-mode – mm)	47 ± 4,9

Fractional shortening (%)	38 ± 7,2

Interventricular septum thickness (mm)	8,9 ± 1,4

Posterior wall thickness (mm)	8 ± 1,5

Right ventricle area (mm2)	9,94 ± 2,56

Left ventricle area (cm2)	21 ± 5,1

Right atria area (cm2)	9,47 ± 3,1

Left atria area (cm2)	12,64 ± 3,2

Tricuspid annular plane systolic excursion (mm)	22 ± 3,7

Inferior vena cava diameter (mm)	15,8 ± 3,6

**Figure 1:**
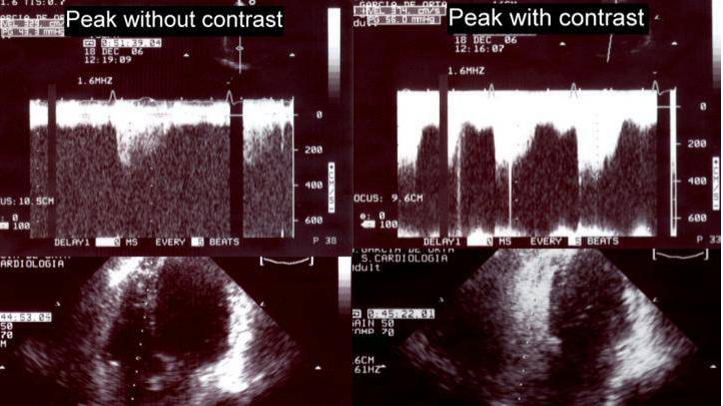
Continuous wave Doppler signal of the tricuspid regurgitant jet at peak exercise, before and after the injection of contrast.

**Figure 2:**
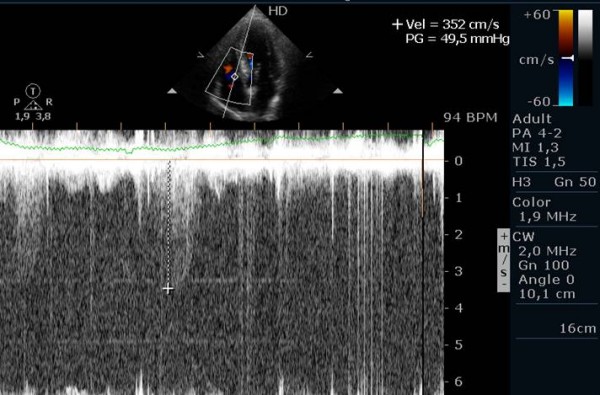
Continuous wave Doppler signal of the tricuspid regurgitant jet at peak exercise, before the injection of contrast.

**Figure 3:**
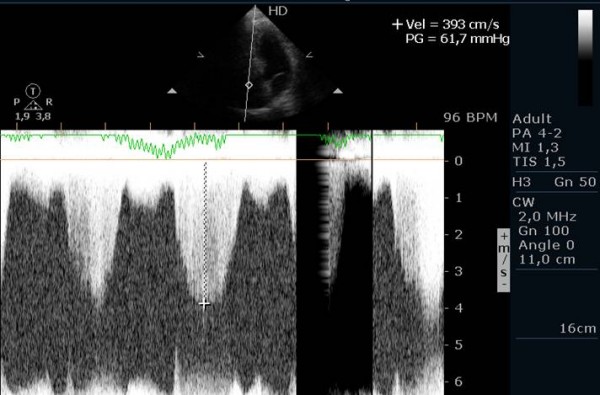
Continuous wave Doppler signal of the tricuspid regurgitant jet at peak exercise, after the injection of contrast.

The quality of Doppler signal was improved in twenty-six patients, deteriorated in five patients and maintained in six patients, with the use of contrast (Figure 1, Figure 2 and Figure 3).

In one patient, a ventricular septal defect was diagnosed (additional file 4).

In six patients, a Doppler signal was only obtained with the use of contrast.

In fifteen patients, a RV/RAg superior to 40 mmHg was only obtained with the use of contrast. Of these, nine have already been submitted to right heart cathetherization, that confirmed the diagnosis of pulmonary hypertension in five of them (56%).

**Figure 4:**
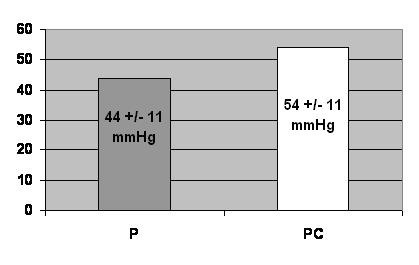
Comparison between RV/RA gradients at peak exercise, without contrast (P), and after contrast administration (PC).

The mean value of RV/RA gradients at peak exercise, without contrast, was 44 ± 11 mmHg and after contrast administration was 54 ± 11 mmHg, p = 0,001. (Figure 4).

## Discussion and conclusion

This method is feasible and applicable in a large number of patients, which is an important characteristic for its use as a screening test.

As occurred in previously published studies, the use of contrast improved the tricuspid regurgitation jet Doppler signal in the vast majority of patients. In some of them, a signal was only obtained after the administration of contrast.

Furthermore, RV/RA gradients obtained at peak exercise are significantly higher with the use of contrast. In the patients with RV/RA gradients superior to 40 mmHg only after the administration of contrast, there seems to be a significant number of false positives.

The number of false positives can possibly be diminished by the use of a lower cut-off value, but the meaning of the difference between peak RV/RA gradients before and after administration of contrast should be evaluated in a higher number of patients submitted to right hear catheterization.

One of the limitations of the method, at this point, is the absence of a clear definition of threshold values.

In conclusion, treadmill exercise echocardiography with contrast seems to have relevant clinical value in the screening and diagnosis of pulmonary arterial hypertension.

Furthermore, it is once again demonstrated that a low cost "home-made" contrast can have a high diagnostic yield, with security advantages when compared to other contrast more expensive and less safe (FDA black box warning on ultrasound contrast media).

The utilization of contrast should probably be limited to the patients with very poor tricuspid regurgitation jet signal, to obviate the apparently high number of false positives results. The augmentation of Doppler signal with contrast, in patients that already have a good quality one, can generate significantly higher RV/RA gradients, including values superior to 40 mmHg, without a clear clinical meaning. So, we think that contrast should be an aid to obtain a measurable RV/RA gradient, but should not be used routinely in all patients submitted to this exam.

## Competing interests

The authors declare that they have no competing interests.

## Authors' contributions

LRL performed exercise echocardiography, reviewed literature and wrote the manuscript. CC performed exercise echocardiography, made clinical assessment of the patients, participate in drafting, and revised the manuscript for important intellectual content. MJL, RM, SA, ARA were responsible for clinical assessment of the patients and revised the manuscript for important intellectual content. AC was responsible for recruitment and clinical assessment of rheumatology patients. MC gave final approval for the manuscript. All authors read and approved the final manuscript.

## Supplementary Material

Additional file 1 **Acquisition of bidimensional echocardiographic images during treadmill exercise: preparing alignment of continuous wave Doppler with the tricuspid regurgitant jet.** The data provided represent the alignment of continuous wave Doppler with the tricuspid regurgitant jet, during acquisition of bidimensional echocardiographic images during treadmill exercise. Click here for file

Additional file 2 **Continuous wave Doppler signal of the tricuspid regurgitant jet, obtained during treadmill exercise.** The data provided represent the continuous wave Doppler signal of the tricuspid regurgitant jet, obtained during treadmill exercise. Click here for file

Additional file 3 **Injection of the agitated saline with blood contrast, filling the right cavities.** The data provided represent the right cavities filled with agitated saline with blood contrast after injection. Click here for file

Additional file 4 **Ventricular septal defect, demonstrated by the injection of contrast in one of our patients.** The data provided represent the ventricular septal defect, demonstrated by the injection of contrast in one of our patients. Click here for file
